# Experiences of Undocumented Parents Reuniting with Children Who Entered the United States as Unaccompanied Minors

**DOI:** 10.3390/ijerph20054496

**Published:** 2023-03-03

**Authors:** Maryam Rafieifar, Miriam Potocky, Hui Huang, Richard L. Beaulaurier, Sloan Bruan Lorenzini

**Affiliations:** 1 Department of Social Work and Child Advocacy, Montclair State University, Montclair, NJ 07043, USA; 2 School of Social Work, Florida International University, Miami, FL 33199, USA; 3 School of Social Work, University of Texas in Arlington, Arlington, TX 76019, USA

**Keywords:** unaccompanied migrant children, immigration, community-based organization, family separation, family reunification

## Abstract

In 2021, the United States saw an exponential influx of unaccompanied migrant children crossing the U.S.–Mexico border. Upon apprehension at the border, unaccompanied children are placed in the Office of Refugee Resettlement (ORR) temporary shelter facilities. The ORR is responsible for locating, vetting, and releasing the children to their family, guardians, or a suitable sponsor. Undocumented parents seeking reunification may fear cross-examination and background checks. This study aimed to explore the experiences of undocumented families reunified with their children with the help of a community-based organization (CBO). A collective case study method was used to collect qualitative data from seven parents. Respondent parents expressed their rationale for allowing their children to cross the U.S.–Mexico border, their experience with the ORR, and the reasons they pursued community-based guidance. The results document the depth of trauma and difficulties parents of unaccompanied migrant children face with American service providers. It is recommended that immigration-related government agencies form relationships with culturally diverse organizations that are trusted by immigrant communities.

## 1. Introduction

The global climate crisis, inequity, violence, and limited resources have worsened many people’s living conditions, exacerbating forced migration [1]. As a result, the number of displaced people achieved a record high of 89.4 million in 2020, and children below 18 make up more than half of this population [1]. Unaccompanied migrant children (UMC) are youth under 18 who migrate from their homelands to another country without a legal caregiver’s presence [2]. Forced migration profoundly impacts the health and well-being of children. Among displaced children, UMC are the most disadvantaged as they face unique challenges that intensify the negative impacts of the trauma experienced before, during, and after the migration [2]. 

Increasing numbers of UMC from Central America are fleeing their countries to the United States. In 2021, over 120,000 UMC who crossed the U.S.–Mexico border were released to parents or sponsors (the highest amount in a decade), and the Office of Refugee Resettlement (ORR) shelter facilities became overwhelmed [3]. A confluence of a lack of shelter capacity and complications from the COVID-19 pandemic intensified and increased the barriers to family reunification [4]. The ORR family reunification screening process requires adults to apply to receive the child, even their own child, and to comply with a rigorous screening process. The adult applicant must undergo a criminal background check, an investigation for child abuse/neglect, and a formal interview. Undocumented parents seeking reunification may fear cross-examination and probative inquiry, which may make them reluctant for reasons related to their own immigration history and status. Parents can, however, secure aid from a community-based organization (CBO) to satisfy reunification demands. This study aimed to explore the experiences of undocumented families reunified with their children with the help of a CBO. 

### 1.1. ORR Procedure

Once apprehended at the border, all immigrants, including UMC, are transported to the Department of Homeland Security (DHS) Customs and Border Protection (CBP) Processing Centers. After a child is determined to be unaccompanied, the DHS refers a child to ORR within 72 h of apprehension [4]. Since 2012, over 350,000 have been referred to ORR, with a drastic increase of 77% in 2021 [3] (from over 69,000 in 2019 to over 122,000 in 2021). 

Unaccompanied children are generally placed in an ORR-funded private shelter, although they remain in ORR custody until they are released to an adult caregiver in the United States, are repatriated to their home country, or obtain legal status. The average time in ORR care in 2021 was 37 days [5]. Around 90 percent of children are reunified with a parent, family member, or another qualified sponsor in the Unites States. Nearly one percent are referred to the Long-Term Foster Care program, and ten percent are sent back to their home country [6]. 

ORR case managers verify the sponsor’s identity, verify their relationship to the child, and confirm the sponsor’s ability to care for and provide for the well-being and safety of the child. The potential sponsor is also required to undergo background checks and complete a sponsor assessment process. 

Once vetted, ORR makes the final decision regarding the placement of a UMC with a sponsor [7]. The ORR also determines if the UMC qualifies for post-release services. 

### 1.2. Literature Review 

UMC leave their countries for various reasons, including fleeing violence, poverty, and unemployment [8]. In 2021, over 90 percent of these youth came from Honduras, Guatemala, and El Salvador [9]. In all three countries, violence is widespread in urban and rural areas [10]. In a 2020 study by the United Nations, 30 percent of unaccompanied children from Central America noted violence (in its many different types, including gang violence) as a reason for their migration [11]. Sometimes, UMC may migrate ahead of their families or follow their parents who have already migrated to the United States [12]. Several studies found that most migrant children are separated from either or both parents during the migration process [13,14,15]. 

A few scholars have documented the unique needs of UMC and the profound impacts of migration trauma and prolonged family separation on them. For example, Linton and colleagues [8] use case-based examples to demonstrate the trauma-related needs of unaccompanied children pre-migration, during migration, upon arrival, and post-release in the communities. Similarly, Berger Cardoso and colleagues [16] used an ecological risk and resiliency model to underscore the needs of UMC during different migration stages, including legal, physical, mental health, education, and safety needs. A qualitative study of 30 UMC reunified with their parents demonstrated that reconnecting with parents after a long separation was a problem for the youth, leading to isolation, loneliness among children, and disruption of traditional family roles [17]. Another qualitative study with 42 UMC found that protracted separation was distressing and multifaceted, with many developmental implications for the youth [18]. These studies recommend providing developmentally appropriate and culturally responsive care to foster healthy adjustment among UMC. 

UMC may experience a wide range of mental health issues. For example, one study found elevated rates of depression, post-traumatic stress disorder, and anxiety in a sample of 24 UMC from Central America [19]. Some researchers in other countries (e.g., in the United Kingdom [20] and Norway [21]) have attributed this increased risk of mental health disorders among UMC to violence before or during migration, dangerous migratory journeys, family separation, and stressful experiences upon arrival. In the United States, a longitudinal study found that immigrant youth who had experienced separation from their parents were more likely to be depressed [22]. Suárez-Orozco and colleagues [23] explain that, depending on a child’s developmental age, the negative impacts of parental separation may include emotional withdrawal, feelings of abandonment, depression, anxiety, and externalizing behaviors. A cross-sectional survey conducted in 2019 of 851 UMC showed that older UMC and female UMC have higher odds of mental health services as a primary need [24]. 

UMC’s access to legal services post-release is another challenge. While in ORR custody, UMC received some legal services, including legal screenings and direct representation; post-release, some children received ORR-funded representation [4]. However, such legal representation was not guaranteed. Most UMC did not receive ORR-funded legal representation [4,25]. Moreover, the fast-track court hearings upon their arrival often negatively impact opportunities for developing a legal claim post-release [19]. A qualitative study of 20 UMC in the Chicago metropolitan area found that there were more limited legal protection options for older children in ORR custody who did not have a sponsor and were nearing 18 years old [25]. This study also found a lack of certainty associated with pending immigration cases, lack of support for recently reunified families, and fear among parents and sponsors related to their immigration status as other legal factors impacting permanency among UMC. Crea and colleagues investigated child welfare outcomes of safety, permanency, and well-being among UMC in a qualitative study [26]. They suggested that, for the UMC population, safety should include the provision of a safe place, both physically and emotionally, to process their prior adverse migration-related experiences. These authors also highlighted that permanency should be examined in terms of obtaining legal immigration status in addition to family reunification options and placement types. The authors further suggested that well-being should incorporate cultural integration and address migration-related trauma.

There is a dearth of knowledge about the experiences of children and families during the reunification screening process (while children are in a CBP processing center and, after that, in the care of ORR shelters). Linton and colleagues [8] reviewed potential barriers UMC might face while in CBP processing centers and ORR shelters before reuniting with their families or receiving immigration relief. They introduced a community-based example that integrates legal and medical care as a model of cross-sector partnerships to mitigate stress and build resilience among UMC. Similarly, the current study aims to extend the knowledge about the experience of families during the screening processes and the role of CBOs in filling the service gap. 

### 1.3. Conceptual Framework

Two theoretical frameworks guided this study: ambiguous loss theory and the conservation of resources theory of stress. 

Ambiguous loss theory [27] is about a type of loss that is unclear and incomplete. Family separation resulting from migration can be characterized as an ambiguous loss because it can be a source of guilt topped with uncertainty about the future outcome [28]. Based on this theory, people sense ambiguous loss when they are physically absent but emotionally present. This can explain the feeling of the parents who migrate to the United States and leave their children behind. Falicov explains that, when individuals migrate separately from their families, they deal with their ambiguous loss in different ways [29]. They often try to reciprocate their absence by money remittances, phone calls, trips back home, and encouraging their other family members to migrate. These practices allow individuals to feel that such separation is provisional rather than permanent. 

The conservation of resources theory’s basic principle is that people strive to obtain or retain resources [30]. According to this theory, stress occurs when people are threatened with losing resources, actually lose resources, or fail to obtain resources. Immigrants experience resource losses, including their homes, family, social support systems, and employment. In addition, they may also experience lower social status in the host country, a decline in their income, and low proficiency in communication. As a result, their self-esteem may also be challenged. A sense of loss may also occur when the migrant’s efforts and investment do not lead to an expected resource gain. This disappointment may also be connected to their feelings of being misled about opportunities in the host country [31]. The conservation of resources theory emphasizes the importance of social support. People with strong social support systems can rely on others to boost resources or alleviate stressful situations to regain resources. However, particularly in under-resourced immigrant communities, the immediate circle of social support may not always be capable of responding to all of their needs. Often, they need to reach further out to individuals or organizations with the resources they need. This can be complex and confusing and raise major issues related to trust, particularly for immigrants with dubious legal status. 

## 2. Materials and Methods

This qualitative study employed a collective case study approach to explore the reunification of undocumented immigrants with their children with the assistance of a CBO. Case study is an appropriate research design for an in-depth examination and analysis of a unique phenomenon [32,33]. This case study was collective in design as it aggregated results across participants. 

The research questions driving this study included the following: (1) What was the experience of parents who had been separated from their children as a result of immigration policies and issues? and (2) Why did the parents reach out to the community organization to be reunified with their children? 

### 2.1. Procedure 

This study was a part of a larger study that used a grounded theory approach to explore factors and processes contributing to undocumented parents’ decisions to delegate guardianship of children to nonrelative individuals, with the primary focus on their experiences of seeking guardianship for their U.S.-citizen children. The study was carried out in an immigrant-populous city in the United States (Miami, FL, USA), and the sample was recruited at a CBO. The CBO identified potential subjects for the study, and interested parents were referred to the researchers. The participants provided consent for participation, and IRB approval was granted by the researchers’ university. 

### 2.2. Participants 

For the parent study, a purposive sampling strategy [34] was used to select the undocumented immigrants with at least one U.S.-citizen child under the age of 18 who made arrangements for delegating guardianship of the children to a third party. The total sample of the parent study included 27 individuals. Parents who met the selection criteria were identified by staff of the CBO, where they received services that included help with paperwork, food and clothing, and legal assistance. Potential respondents expressing interest in the study were asked to choose pseudonyms before contacting the research team to protect their anonymity. Respondents who were interviewed received $20 for participation. All but nine of the individuals who were identified as meeting the selection criteria agreed to participate in the study. The anonymous nature of participant recruitment made it impossible to collect demographic information rationales from potential respondents who declined to participate. 

The current study utilized a sub-sample recruited from the parent study. Seven parents were identified who, in addition to meeting the selection criteria for the parent study, had also been separated from their foreign-born children as a result of immigration enforcement actions. With the assistance of the CBO, these parents were eventually reunited with the children from whom they had been separated. The demographics of these participants are shown in Table 1.

### 2.3. Data Collection 

Data were collected by conducting in-depth interviews with respondents of approximately 45 min in length. The interviews took place between February and September 2021. All interviews with the parents were conducted in Spanish by a bilingual master’s-level social worker. At the beginning of the session, a consent statement was read to the participants in their native language, and verbal consent was obtained. Research participants were reminded that they could withdraw from the study at any time without fear of reprisal. They were also assured that the researchers and the interviewer did not know their real names and would only know their pseudonyms to guarantee their anonymity and privacy [32]. The study utilized a semi-structured interview schedule to facilitate a casual conversation format [35]. The interview schedule was drafted by one of the authors and finalized after consultation and feedback with experts on the population under study and qualitative interviewing. In addition, CBO staff reviewed the interview schedule and provided feedback. This was particularly helpful as these staff were both familiar with and culturally similar to the participants. The interview schedule was divided into four topics: The Undocumented Experience; Determining Guardianship; Communicating with Children; and Dreams and Aspirations. The protocol was further broken down into questions and prompts. As this article focuses on the experiences of undocumented parents reuniting with children who entered the United States as unaccompanied minors, the questions and prompts on the first topic are listed in Table 2. 

Interviewers were trained to ask the questions on the interview schedule in their own words and in a natural, conversational style. Prompts were optional, to be used if the participant’s answer did not cover issues of interest or to flesh out responses. All interviews were audio-recorded and transcribed verbatim in the original language (Spanish). The transcriptions were performed by a Spanish-speaking volunteer who was not involved in the interview process. Final transcriptions were checked against the recording to ensure accuracy. Subsequently, a Spanish-to-English speaker translated the transcriptions into English. To ensure the quality of the translation, the English translation was checked by another bilingual person who was not involved in the transcription or translation. This process was carried out to ensure that the Spanish transcriptions had been accurately translated into English.

### 2.4. Data Analysis

Computer-assisted qualitative data analysis software (CAQDAS; ATLAS.ti [36]) was used to organize and assist in analyzing the translated texts. Each English transcript was loaded into ATLAS.ti and read twice before coding began. The authors first generated a list of a priori codes based on the literature relevant to the research questions. Those a priori codes that could not be linked to actual quotations were dropped from the analysis. Most codes used in the analysis were generated through open coding. Open coding involved line-by-line reading of the transcripts with the object of assigning codes to passages of text where they occurred to produce as many different categories of response as possible [37,38]. This process involved iterative reading of the transcripts until saturation was reached and no more codes could be attached to the text. The constant comparison method was used to make sure that codes were consistently applied throughout the analysis. As new codes were found, previously reviewed transcripts were examined for evidence of newly emerging concepts and codes. Negative case analysis was also used to verify key findings [39]. This mainly included searching for data elements that did not support or contradicted patterns emerging from the analysis. Liberal use of memos attached to codes and other elements of the analysis were used to leave an “audit trail” of decisions taken throughout coding [39,40]. Grounded codes (codes attached to passages of text) were examined for patterns that could be grouped into higher-level, more abstract categories and higher-level codes. These codes were linked to grounded codes. Relationships between grounded and emerging higher-level codes were explored [41]. The “network” function in ATLAS.ti was used to create relationship maps to further develop and visualize emergent concepts and themes [42]. A benefit of this approach is that it allowed researchers to document and verify the linkages between coded passages of text through higher-level codes, concepts, and themes that emerged from the analysis. Thus, even the most abstract concepts and themes could be traced back to participant responses.

## 3. Results

Seven participants were interviewed between February and September of 2021. Except for one man who participated in the interview with his wife, all other participants were women. All participants were Central American or Mexican. One participant reported that he did not have an occupation, and those who were employed received meager compensation for low-skilled services in industries such as agriculture (2), housekeeping (2), construction (1), and restaurant food services (1). Their separated children were all female, between 7 and 11 years old. All participants had been separated from their children. Five had been reunited and two others were in the process of reuniting their families. Most participants (5) still had at least one child residing in their countries of origin and stated that they were looking for an opportunity for those children to join them in the United States. Two participants reported that they had successfully reunited the whole family.

### 3.1. Voluntary Separation in Native Country

All participants had experienced family separation because of forced migration. They decided to leave their home countries for violence, livelihood difficulties, and lack of jobs. However, they indicated that migration through irregular channels to the United States was both “expensive” and “dangerous.” Often, the families assumed the risk and paid strangers (coyotes) to help them cross the border. According to the families, the coyotes would ask for a significant amount to be paid for each person. Many of them had to sell their possessions or borrow money to be able to cross the border. One interviewee said they had to pay $3500 per child to the coyotes to get them across the border. Another woman said she had to mortgage a piece of land they had in Guatemala to take a loan to pay the coyotes. Expense is why they all stated that they decided to migrate in stages, leaving the children behind in the care of extended family members and having them join the parents once “they settled” and “saved some money”.

All participants indicated that they worked and sent money to their children and sometimes grandparents and siblings. They talked about the difficulties of the family separation for themselves and their children left behind. The Guatemalan couple who left their three children with their grandparents said the following:

*The children feel lonely. When we talk on the phone, they say, “we would like to be together. It is good that you send us a little money, we use everything, but we need your warmth. Sure, we want to have your love.” We need the family, we lack that bond, but unfortunately, it is …expensive to come here together.*

It is often difficult for children to understand why their parents choose to migrate without them. One parent said, “I have two boys who live with my parents. One of them worries me a lot because he does not understand why I left them”. The parents also suffer significantly because they “cannot stop blaming themselves”. Most respondents became very emotional when talking about their children who were left behind. One participant said, “this is a matter of stress; I understand the pain I have. I feel stressed”. The other said she had headaches whenever she talked to her children back home. Another 30-year-old woman said she developed diabetes after she came to the United States. 

### 3.2. Parental Rationale for Child Migration 

All parents stated that they had plans to bring their children to the United States when possible. The planning included saving money, finding a reliable adult who could accompany the children, and waiting for a time when they were more likely to succeed in entering the United States. Two parents mentioned that, when the new president came to office in the United States, they hoped the policies would be less hostile towards immigrants. “I hope this president is better than the last one and that he supports and continues to support all immigrants”. 

Two other parents said that unexpected events in their native country threatened their children’s safety, causing them to consider the child’s migration. For example, one participant explained that her daughter was raped. So, she asked her children to leave the country and cross the border to join her in the United States. At the border, the oldest girl, who was 18 years old, was returned to Mexico, and the two younger girls were sent to a temporary shelter. Another parent said she had to take the risk and have her seven-year-old daughter pass the border with a neighbor after her older son suddenly died. She believed the risk of the child remaining in the home country was greater than having her join her mother in the United States. However, the journey also involved risk, as another respondent indicated.

*So, we left our small children, we left four little ones. There is one, who is three years old, three years old! We wanted to bring them here. We screwed up. In Mexico, they were grabbed. I lost money because I paid someone to get them to this side. But in Mexico, there is also an immigration law. So, they grabbed them and returned them to my country again and put a lawsuit on us that said he couldn’t bring them.*

### 3.3. Experience with Bureaucracy

All parents stated that a trusted adult accompanied their children to the border, but at the border, the children were considered unaccompanied and separated from those adults. Once the children entered the country, they were placed in temporary care. Overall, the parents described their experiences in locating their children and getting reunited with them as “difficult”, “painful”, “complicated”, and “anguished”. The parents had two main concerns: not knowing where their children were and fearing the possibility of losing them. 

As the children were apprehended at the border and got separated from the accompanying adult until they were assigned a case worker in temporary care, the parents often did not know where their children were. In cases where there were communications with the immigration enforcement agent, the interviewees did not seem to fully understand the process. For example, one of the interviewees depicted it as follows:

*The only thing I know is that on a Tuesday morning, [my older daughter] called me to let me know that she had been separated from the [other] children and that they were going to remove her…the following Sunday, at about three in the morning, a Border Patrol agent called me and said that the [other] girls were going to be transferred to a youth center.*

Parental fears were exacerbated by lack of access to their children. Once the children were transferred to the ORR temporary shelter, the parents and children were able to communicate. However, the parents were confronted with a new barrier: they had to legally prove parental rights. ORR administration requires parents to provide documentation to release children back into their custody. They all feared that they would lose custody of their children if they could not fulfill the government’s requirements. Here is how a woman from Guatemala describes her devastation:

*My daughter was taken to Michigan and spent two months alone in a shelter. And there, they asked me to do papers and said they would give her up for adoption if I didn’t get the papers.*

Preparing the required paperwork was a great challenge for all of the interviewees. Many families said they had difficulties finding the necessary documents to prove the parenting relationship. For example, one interviewee from Mexico said the following:

*When you come here to this country, you leave everything; you don’t bring any photos or documents… And to start putting everything together, I had to grab the phone and talk to the people to find their photos and documents... at that time, I wanted to die.*

From the apprehension of children at the border to the time they reunify with their parents, parents often need to deal with multiple systems: immigration authorities, ORR staff members, and shelter case managers. All participants were unhappy with the guidance and services they received. Some parents talked about the degrading treatment they had received from immigration authorities. For example, a woman described her encounter with the immigration authorities, “I was treated very badly, worse than an animal. They thought I didn’t understand what they spoke in English, but I did understand, and they didn’t give me a chance to speak”. Some others said they could not communicate with immigration authorities before their children were transferred to a shelter. “When immigration caught her, we did not know where she was for three days…for three days I lived in fear, and then they called from [the shelter]”. Some mentioned frustration with the shelter case managers.

*The social worker that I had, that woman was like having a little knife at her neck, she didn’t cut me, she just hurt me, she hurt me, and I had to do what she said because somehow it was the only way to get my daughter back.*

Another interviewee described her caseworker as a “police officer” who only expected her to “obey”. She said, “I was a robot [to her]… She was like: ‘I tell you this, you do, period. I want this, and I want it now”. A third interviewee whose daughters had been in the shelter for two months related that she

*…told [the caseworker] to repatriate my daughters and that I will go back to Mexico. I told her I didn’t care. This country did not give birth to me. She said, ‘then go away. Go any time you want.’ I said Ah, well, if you want to punish me so that I could never return to this country, I accept it with enthusiasm, but give me my daughters.*

Several participants did not differentiate between government-affiliated agencies. They just talked about the lack of clarity in communications. A participant said, “the government didn’t tell us anything, we didn’t know where she was, we were in this [situation] for two months”. Another one said, “they don’t explain why things are done…they don’t want to explain. We are treated like second-class people”. 

Additionally, language was a barrier and contributed to maltreatment. The Guatemalan participants mentioned indigenous languages as their native language, and Spanish was only their second language. They talked about difficulties they had in understanding the legal requirements. “I don’t understand Spanish much. I speak an indigenous dialect. I did not understand what I needed to sign for my girl, whether it was to give her away or not”. Another participant who had her daughter in a temporary facility for two months had an easily treated condition that became serious because it was not detected or treated.

*She came back very sick because she came back with a significant infection in one tooth. She almost had rotten gums. Then she spent several days with a fever, and since she did not speak English, she was not treated.*

### 3.4. Family Separation within the United States

While migration-related family separation is often voluntary, family separation initiated by U.S. immigration seems to be of another nature. When parents leave their children in their home countries, they usually have a protection plan in place. For example, many parents in this sample left their children with their older siblings or grandparents. They would talk with their children regularly, and they had some feeling of control. In contrast, when children pass the border and enter the U.S. immigration system, parents become apprehensive. This is perhaps because they do not trust the system and fear they could lose their children. One woman who was reunified with her daughter after two months said she “felt very lonely and desperate” when her daughter was with ORR. She explained that before that, even though her daughter was away, she knew she was with her sister and that she would be cared for. 

When discussing family separation within the United States, most participants said that they had feared that their children would “get stuck” in the system and that they would lose them. They did not trust the government. One woman explained that, when her daughters were in ORR custody, her family was suspicious that she would be able to reunite with her children. “They said that the government had taken the girls from me and that they were never going to give them back to me”.

Another woman mentioned that her children were worried about their sister, who was in the shelter. The children asked many questions she could not answer: “They ask me when is she going to come? How will it be? Will she be eating? Why is she not there? They are afraid of losing their sister”. Respondents indicated their children were highly distressed when in the child welfare system. For example, one interviewee said, “we talked on the phone every day when she was in the shelter. She would call me every day and cry every time, saying it was because of her that this happened”. 

### 3.5. Community-Based Services

When asked why parents sought community-based services, the participants provided three different reasons: lack of other alternatives, family and friends’ recommendations, and resourcefulness of the CBO. The main reason for almost all of them was the lack of alternative options. They all mentioned that they “did not know where to go”. Many of them had tried other ways to get in touch with their children but failed. They often did not have family members who could help them, or that “I have a family. I have brothers and sisters. But they are undocumented, just like me. Like I said, we’re here in this country without papers”.

Most participants became aware of the community-based services through their friends; families; and, in two cases, through their church. They trusted the CBO because people they trusted had introduced them.

*We went to [church] to ask for help with our little girl because we didn’t know how to get her out, and there they told us about [the organization]. They said they would help get my daughter out and that [the organization] had helped many children before.*

Three participants said they learned about the CBO because they or their friends and relatives had seen programs about it.

*I have a friend from California who saw them on TV. They were talking about a case similar to mine. He told me that he had found this organization, and he told me, “I spoke to them, they answered me and gave me all the hopes that they can help you, I have their phone number. Talk to them immediately”.*

The participants mentioned that they understood that the CBO staff were resourceful, knew how to navigate the governmental systems, and could provide the support they and their children needed. This was the main reason they trusted the CBO and sought their help. Participants also mentioned that they witnessed how the agency had helped other families. “I saw that they did help many children. They had them living here. She [the agency director] would help children if their parents were deported”. In addition, most participants mentioned the CBO was available and that they could “reach out” to them when they had concerns or questions. “I always talked to [the CBO] so much. They explained everything with patience”. 

One interviewee who was reunited with her two daughters, whose case had been stalled for some time with the child welfare agencies, talked about the help she received from the agency director.

*When I talked to her it was like everything started moving again, like she revived the case. Like the social worker had my document on the desk forgotten, and she came and opened the case... Her involvement changed everything, and I could talk again to the social worker through her.*

Another participant said that the agency

*…helped me get my girl out of immigration and took me to a lawyer. I could talk to the lawyer about my kids, and he helped*

## 4. Discussion

### 4.1. Trauma of Separation

There is a considerable body of literature [17,18,43] indicating that family separation is one of many adversities children and families experience before, during, and after migration, and it negatively impacts individuals’ health and well-being [8,16,43] and the parent–child relationships [15,17]. The findings of the current study are consistent with other studies in this regard. In addition, it provides insights into the different natures of family separation before and after migration. Before migration, family separation was a choice by the parents to leave the oppressive sociopolitical context of their home country; after migration, family separation was not their choice but was forced on them. 

Migration for these families required a Solomon’s choice, not once, but twice. Migration was not seen as optional for these parents, either for reasons of abject poverty or the mortal danger they saw themselves as fleeing. Poverty was the leading reason that parents separated from their children. The expense of both licit and illicit means of entry to the United States made it impossible for all family members to travel together. Undocumented entry into the United States involved dangerous, expensive travel. In many cases, immigrants must cross multiple national borders. Illegal crossings often involve high expenses owing to travel, the need to pay coyotes, and the high risk of theft and victimization along the way. 

For immigrants with little education, little access to financial resources, and limited travel opportunities, the expenses and difficulty of licit and illicit immigration for the whole family were simply insurmountable. Thus, independent travel and family separation were seen as their only option. Similarly, while families were able to assemble enough resources to bring their children to the United States, they were not able to travel with them. Reasons included travel restrictions, documentation status, and expenses. Thus, reunification meant having someone without legal guardianship bring the children, meaning they would enter the United States as unaccompanied minors. 

Like the decision to leave children behind and immigrate to the Unites States, the decision to reunify was fraught with guilt, danger, anxiety, and fear for these parents. The decision to leave children in locations where they were exposed to poverty and violence would have been similarly fraught and was compounded by the knowledge that their children often did not understand why their parents had left them. The choice to leave was a Solomon’s choice. The choice to reunify was another Solomon’s choice. In both decisions, parents are forced to weigh the trauma of separation against the real dangers of trauma, injury, or mortal danger for their separated children.

The results illuminate what undocumented immigrant parents experience when a safe choice is inaccessible. The parents separated from their children experienced various types of loss, including safety, predictability, knowledge of family member status, resources, and culture. These findings are consistent with the ambiguous loss theory because the parents all felt guilt when talking about leaving their children behind. They all mentioned sending money back home and discussed plans to eventually get their children and sometimes their parents to the United States. Permitting their children to pass the border without a legal guardian was an effort to overcome the ambiguous loss. Although the parents voluntarily separated in their native country, this decision was not a good option, but was the lesser of two evils, especially when considered within an oppressive sociopolitical context. The parents could not afford to travel as a family, and this choice was made because it was the most economical and least dangerous option. However, the parents did feel some reassurance when their children lived with other family members in their native land. This may have provided the parents with a temporary sense of control and predictability, thus reducing their stress when they were in the United States. Following the family separation and migratory journeys, parents reported additional fear of loss and disappointment after they arrived in the United States. Many parents expressed the emotional burden endured because their child’s/children’s safety was unknown, and the demands imposed by the ORR caused parents to re-consider that they may lose their children. Furthermore, as a parent is responsible for providing safety, their sense of guilt and shame is strong.

### 4.2. “I’m from the Government, and I Am Here to Help.”

The quote from President Reagan above, which he characterized as “the nine most terrifying words in the English language”, captures the experience of migrants in the sample. Respondents reported only negative experiences in their contact with American bureaucratic processes. ORR does not provide shelter services or other social services. Contracted local agencies funded by ORR render these services. Parents in the sample reported callous treatment by agents of these bureaucracies. While it is often impossible to tell precisely by which agency or in what capacity the personnel was employed, two things are clear. Respondents regarded all of these agency personnel, as well as law enforcement, as essentially the same, and these personnel exacerbated parental fears about losing their children. Respondents mostly had low levels of education and were fleeing abject poverty, violence, political turmoil, or a combination. They often traveled to the United States with few possessions and little understanding of American bureaucratic practices. Thus, the need to produce documentation and the threat of losing their children could be confusing and terrifying. 

One of the limitations of these data is that it is unclear if the perceptions of callousness and the fear with which these respondents regarded ORR and their contractors were fueled by predisposition. Historically, it has not been uncommon for immigrants to fear agents of government bureaucracies or social and health personnel who they believe may be acting in concert with law enforcement. However, these predilections may also be fueled by the political and legal climate at the state and federal levels. There were record deportations under the Obama Administration, and policy under the subsequent Trump administration was famously, and overwhelmingly, anti-immigrant [44,45,46]. It is reasonable to speculate that respondents were already predisposed to fear and mistrust of U.S. government agencies and personnel and that this carried over to the contractors with whom they worked. Additionally, it is likely that the participants had had negative experiences with government agents in their country of origin.

Thus, it is not surprising that respondents may have had a predilection to fear and mistrust personnel that they saw as government agents. It is more interesting that respondents had far more positive things to say about the CBO that they had encountered through familial and community contacts. Respondents indicated that they found the agency by word of mouth from trusted individuals, favorable media coverage, churches frequented by immigrants, and testimony from people in their cultural circle who had worked with the agency. Although not mentioned specifically by these respondents, it may have also helped that the agency director was herself a Central American immigrant, as were many agency staff and volunteers.

### 4.3. Recommendations

What is most evident in the data is that these respondents and their unaccompanied children have experienced difficult and often traumatic experiences in their process of immigration. This partly relates to situations in their home countries that lead to immigration and partly to the journey itself. Clearly, one part also relates to treatment by U.S. immigration officials and staff tasked with helping the children. There is a need for programs targeting parents’ and children’s stress and the impact of trauma experienced due to family separation [47]. Such programs should focus on mitigating uncertainty and working with families to manage the stress of themselves and their children. The National Child Traumatic Stress Network suggests that service providers regularly screen children for exposure to trauma; provide evidence-based, culturally responsive assessment and treatments; focus on protective factors that support resilience in families affected by trauma; establish continuity of care; and address the traumatic experiences of the parents [48]. 

It is also clear that resources that these respondents found through their own networks, communities, and similar cultural groups were perceived as more trustworthy and helpful by these families. Child welfare systems have been called to increase the involvement of community members in identifying and developing services [49]. Although more study is warranted, this study also suggests that government agencies involved in the immigration process need to build relationships with agencies that are more culturally integrated and trusted by immigrant communities. Such agencies present challenges to large federal agencies that are used to dealing with large, mainstream social service agencies that have in place grantsmanship, accounting, professional staff, and economies of scale that make them good contractors. By contrast, agencies that operate in and are well integrated with minority communities are mainly small, often poorly funded, alternative service organizations [50,51,52]. Such organizations typically do not have the capacity to respond to Federal RFPs or contracting opportunities. Rather, to effectively work with such agencies, federal bureaucracies will need to seek them out and provide contracting support [51]. Similar outreach to faith-based communities, such as local churches frequented by immigrants, is recommended. Standard contracting approaches, however, are unlikely to be effective with these community-based entities. Moreover, these results can be used in applied social work, including organizationally streamlining procedures and program development, to design training modules that can decrease trauma experienced by the family.

Finally, although a comprehensive immigration reform might be far-fetched, small policy enhancement measures could go a long way. For example, establishing the Central American Minors (CAM) Refugee/Parole Program allows nationals of El Salvador, Guatemala, and Honduras lawfully present in the United States to bring their children through refugee admission or humanitarian parole [53]. However, such programs do not currently cover undocumented immigrants. Similar policy and legislative measures are suggested to safeguard the rights of UMC. For example, improving the screening and adjudication processes or providing training for governmental staff working with UMC and their families are among the policy measures that could be implemented, especially based on the UN Convention on the Rights of the Child [54]. 

### 4.4. Limitations

Despite the contributions of this study, there are several limitations. First, the researchers did not conduct follow-up interviews or seek additional information or clarification. Undocumented populations are notoriously difficult to interview. As findings indicate, in many cases, they have good reason to be leery of officials wishing to interview them. To gain their trust and honor their desire not to be identified, interviews were limited to being conducted anonymously and in a single location they found safe. This made it impossible to conduct follow-up interviews or to seek additional information or clarification.

Second, this study was a byproduct of a parent study focusing on undocumented parents seeking guardianship for their U.S.-citizen children. The current study utilized a sub-sample of the parent study. Cautious interpretation is necessary owing to the small sample size of the parent study and the fact that all participants were recruited in a single agency in a single geographic location. Moreover, the primary focus of the parent study was on undocumented parents seeking guardianship for their U.S.-citizen children. In the course of conducting that study, it was discovered that a sub-sample of these parents had reunited with children from whom they had been separated in their immigration process. This unique and serendipitous sample provided interesting insights that have not previously appeared in the literature. However, studies should be designed that focus directly on this population.

Third, the sample size is small, which limits its generalizability. Primary data collection occurred during the height of the COVID-19 pandemic. This posed significant challenges to data collection and contributed to the small sample size. As indicated, most respondents had limited economic resources. This included limited digital literacy and a lack of access to digital communications technology, which might have facilitated additional participation of potential respondents.

## 5. Conclusions

Disadvantaged communities cannot always plan their migration as a cohesive family unit. In addition to migration challenges, they face other adversities due to a lack of choice and family cohesion. As a result, families of UMC sometimes have no choice but to permit their children to migrate without a legal caregiver. Parents of UMC often left their children to travel with others because of the risk associated with their own immigration and their limited resources. Traveling without their parents, UMC may experience trauma during migration and upon arrival in the United States because they are placed in shelters unknown to them. Undocumented families often face challenges with family reunification. These adverse experiences profoundly impact the children’s and their families’ health and well-being The findings suggest that government entities involved in immigration should establish connections with organizations that are culturally diverse and have the trust of immigrant populations.

## Figures and Tables

**Table 1:** Demographic characteristics of participants.

Demographic Characteristic	
	n
Gender	
Female	6
Male	1
Country of Origin	
Guatemala	4
Mexico	2
El Salvador	1
Education	
Primary or less	3
Middle school education	1
High school	3
	Range
Age	29–41

**Table 2:** Questions and prompts for the topic Undocumented Experience.

Question: Why did you decide to come to the US?
Prompts: family? Money?A new life? Security?
Question: What is it like to live without papers? Question: How does it impact your daily life?
Prompts: Driving routes? School Routines? Avoiding field trips?Avoiding travel?
Question: What is the most difficult thing about being undocumented?
Question: Imagine having papers: how would life be different?

## Data Availability

The data are not publicly available while manuscripts are under development and until they can be redacted to fully protect the confidentiality of respondents.
